# Effects of maternal mild zinc deficiency and different ways of zinc supplementation for offspring on learning and memory

**DOI:** 10.3402/fnr.v60.29467

**Published:** 2016-01-29

**Authors:** Xiaogang Yu, Weiwei Chen, Zhenzhen Wei, Tianhong Ren, Xin Yang, Xiaodan Yu

**Affiliations:** MOE-Shanghai Key Laboratory of Children's Environmental Health, Xinhua Hospital affiliated to Shanghai Jiao Tong University School of Medicine, Shanghai, China

**Keywords:** zinc deficiency, zinc supplementation, rat, learning and memory

## Abstract

**Background:**

The effect of different ways of zinc supplementation on spatial learning and memory remains unclear.

**Objectives:**

This study aims to assess the effectiveness of two ways of zinc supplementation – oral use and intravenous transfusion – in zinc-deficient offspring rats on learning and memory.

**Design:**

Rats were randomly divided into six groups on the first day of pregnancy (*n=*12): control (CO), pair fed (PF), zinc deprived (ZD), oral zinc supplementation (OZS), injection zinc supplementation (IZS), and injection control. The offspring's spatial learning and memory were tested at postnatal day 35 using Morris water maze (MWM). Maternal rats’ serum zinc was measured at postnatal day 21, while pups’ serum zinc was measured at postnatal day 35.

**Results:**

Compared with the CO and PF groups, pups in ZD group spent more time finding the latent platform and swam longer distances (*p<*0.05). Compared with ZD groups, pups in OZS group significantly decreased the time used for finding the platform and the swimming distance (*p<*0.05) and were similar to that of CO and PF groups (*p>*0.05). However, compared with ZD groups, pups in IZS did not show any improvement in the indexes of MWM (*p>*0.05) although their zinc serum concentration increased significantly (*p<*0.05).

**Conclusions:**

These results indicate that mild zinc deficiency during pregnancy and lactation leads to the impairment of learning and memory function in offspring, and that OZS, instead of intravenous transfusion zinc supplementation, can recover the impairment of spatial learning and memory function.

Severe zinc deficiency is considered to be rare, while mild or moderate zinc deficiency is more widespread (1). It is estimated that 82% of pregnant women worldwide have a zinc intake lower than the recommended dietary intake, and this may approach 100% in developing countries (2). Free zinc (Zn^2+^), one of the most abundant divalent metal ions in the central nervous system, is mainly stored in the synaptic vesicles of excitatory synapses, particularly the synaptic terminals of hippocampal mossy fibers, which is important for maintaining cognitive function (3, 4). Free zinc is coreleased with neurotransmitters in response to synaptic activity (5, 6), and it is known to modulate postsynaptic neurotransmitter receptor activity. Free zinc is important for myelination and for the release of the neurotransmitters gamma-aminobutyric acid and glutamate, which are key modulators of neuronal excitability (7, 8). The developing nervous system is a prime target for the disruptive effects of zinc deficiency, as the brain undergoes its most rapid period of maturation during fetal life. Studies have shown a correlation between maternal zinc status and neonatal and infant behavior and cognitive function (9). Some studies have reported that dietary zinc deficiency can cause a decrease in zinc concentration in the hippocampus (10). Few intervention studies in human populations suggested that improving maternal zinc status through prenatal supplementation might improve fetal neurobehavioral development (9). However, the limited studies on the effects of zinc supplementation on cognitive recovery in zinc-deprived (ZD) animal offspring have reported conflicting results (11, 12). Thus, further research is required for definitive conclusions to be drawn.

In clinical practice, oral zinc 1–2 mg/kg/day is commonly used for treating zinc-deficient children (13, 14). Studies of parenteral zinc supplementation were limited and the majority having been done in populations known to have increased zinc losses, such as burn, major trauma, and cardiac surgery (15–17). In some special cases where absorption via the oral route is impaired in the setting of critical illness and long-term intravenous alimentation, the suggested zinc dose by vein is 300–500 µg zinc/kg/day (18, 19). There are some reports about the impact of OZS for zinc-deficient rats on cognition (11, 20), but little work has been done on the effects of non-oral zinc supplementation on the cognition of the offspring deprived of zinc during gestation and lactation. In the present study, we have explored the effects of two ways of zinc supplementation on the cognition of the offspring rats deprived of zinc during gestation and lactation.

## Materials and methods

### Diets

Rats were fed an egg white protein–based semipurified experimental diet based on the AIN-93 recommendations (21). The experimental diets differed only in zinc content, containing zinc 25 µg/g diet (control diet), 2 µg/g diet (mild zinc-deficient diet) as zinc carbonate, which was confirmed by atomic absorption spectrophotometry (Thermo M6, Waltham, USA).

### Rats

**Fig. 1:**
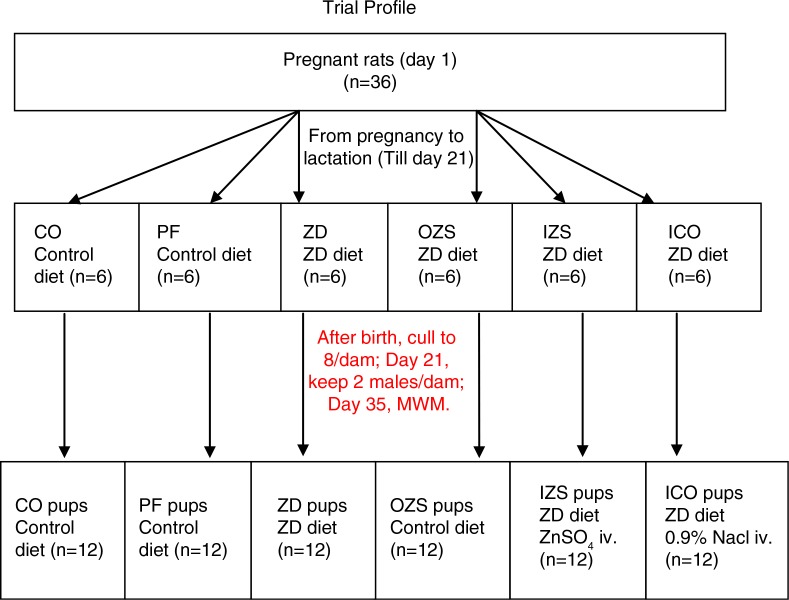
Trial profile demonstrating number of dams and pups in each group that were fed with Zn-deficient diet (Zinc 2 µg/g) or control diet (Zinc 25 µg/g). CO=control; PF=pair fed; ZD=zinc deprived; OZS=oral zinc supplementation; IZS=injection zinc supplementation; ICO=injection control.

This study complied with the Guide for the Use and Care of Laboratory Rats and was administered under the auspices of Animal Resource Services of Xinhua Hospital affiliated to the Medical School of Shanghai Jiao Tong University, which had been accredited by the Chinese Association for the Accreditation of Laboratory Animal Care. Thirty-six virgin Sprague Dawley rats (220–250 g) were obtained from a commercial source (Bikei Animal Company, Shanghai, China). The rats were maintained in stainless steel hanging cages in a temperature-controlled facility with a 12-h dark–light cycle. After consumption of a standard non-purified diet (Bikei Animal Dietary, Shanghai, China) for a 5-d acclimatization period, the rats were mated to obtain offspring. Rats were randomly divided into six groups from the first day of pregnancy: control (CO), pair fed (PF), zinc deprived (ZD), oral zinc supplementation (OZS), injection zinc supplementation (IZS), and injection control (ICO) (Fig. 1). Each group had six pregnant rats. Both the CO group and the PF group were fed the control diet (Zinc 25 µg/g) with the PF group receiving only the daily average weight of food eaten by their ZD group. The ZD, OZS, IZS, and ICO groups were continuously fed with the zinc-deficient diet (Zinc 2 µg/g) from pregnancy to lactation. After weaning (day 21), two male pups with similar weight were kept with each dam, and thus each group had 12 male pups. Pups in the CO and PF groups continued to be fed with the control diet and pups in the ZD group with the zinc-deficient diet. The OZS pups proceeded with the control diet. The IZS pups were given 1 ml 0.9% NaCl solution containing 2.5 mg ZnSO_4_ (Shanghai Chemical reagent Company, Shanghai, China) by intravenous transfusion once every day, while the ICO pups, which served as injection control, were injected with 1 ml 0.9% NaCl solution without ZnSO_4_ in the same way as IZS group. Meanwhile, both the IZS group and the ICO group continued to be fed with the zinc-deficient diet. All six groups were fed deionized water ad libitum (Fig. 1) (Table 1). Diet intakes were measured every day and body weights were measured twice per week. When pups were 35 days old, the experiments described below were conducted.

**Table 1:** Experiment zinc supplementation design of six groups

Group	Pregnancy Zn	Lactation Zn (till day 21)	Weaning Zn (days 21–35)
CO (controls)	25 µg Zn/g feed	25 µg Zn/g feed	25 µg Zn/g feed
PF (pair fed)	25 µg Zn/g feed, but the amount of food is the same as in the ZD group	25 µg Zn/g feed, but the amount of food is same as in the ZD group	25 µg Zn/g feed, but the amount of food is same as in the ZD group
ZD (Zn deficient)	2 µg Zn/g feed	2 µg Zn/g feed	2 µg Zn/g feed
OZS (Oral Zn)	2 µg Zn/g feed	2 µg Zn/g feed	25 µg Zn/g feed
IZS (intravenous Zn)	2 µg Zn/g feed	2 µg Zn/g feed	Zn-deficient diet, 2.5 mg ZnSO_4_ intravenously
ICO (intravenous control)	2 µg Zn/g feed	2 µg Zn/g feed	Zn-deficient diet Injection without Zn

### Morris water maze

Beginning on postnatal day 35, the rats received 4 days of training to test their capacity for learning and memory acquisition using a Morris water maze (MWM), as previously described (22). Briefly, for the place navigation test (spatial learning acquisition), each animal was subjected to two trials per day for 4 consecutive days. Each trial consisted of placing the rat in water so that it faced the wall of the pool at one of four starting locations (North, East, South, and West) in random order. The rats were allowed to search for the platform for a maximum of 120 sec. If an animal did not find the platform in 120 sec, it was gently lifted up and placed onto the platform for 20 sec before being returned to the cage. The escape latency (the duration before finding the platform) and swim paths were automatically recorded by a video-computer system. The escape latency, path length, and swim speed were recorded as indexes of learning and memory capacity.

### Sample collection and analysis

After weaning (day 21), maternal blood was drawn from the right orbital vein via a syringe for measuring the serum zinc level. At the end of the MWM test, offspring rats were anesthetized via short inhalation of ether; blood was then drawn from the right orbital vein via a syringe for measuring the serum zinc level. The blood samples were centrifuged (3,000 rpm for 5 min) (Labofuge 400r, Heraeus Instrument, Hanau, Germany) and serum samples were kept at 85°C. The zinc level in the serum was determined by an atomic absorption spectrophotometer (Thermo M6, Waltham, USA).

### Statistical analysis

All data for performance as well as body weight and serum zinc were expressed as the mean±standard deviation. Data were analyzed to test specific hypotheses using SPSS 17.0. Statistical analysis was performed by means of one-way ANOVA. Tukey's honest significant difference test was used to assess any significant differences in the results. A *p*-value of 0.05 for the various outcomes was considered statistically significant.

## Results

### Zinc deficiency affected the serum zinc concentration and growth of the rats

In the ZD group, maternal rats had growth retardation after being fed a zinc-deficient diet for about 8–9 days (Table 2), and then showed zinc deficiency symptoms including diarrhea and indifference (compared with CO and PF groups). After weaning (day 21), concentration of maternal serum zinc in ZD group was significantly lower (compared with PF and CO, *p<*0.05) (Table 2). In addition, maternal serum zinc concentrations between CO and PF groups were not significantly different (*p>*0.05) (Table 2). These data showed that the model for zinc deficiency was successful. Pups in the ZD group had lower birth weight and were late to open their eyes, while some of them had eye infections, bad appetite, agitation, indifference, and growth retardation. On day 35, pups in the ZD group had significantly lower serum concentrations of zinc and lower weight compared with the PF and CO groups (*p<*0.05) (Table 2). Differences in serum zinc concentration between CO and PF groups were not significant (*p>*0.05), while differences in weight gain between CO and PF groups were significant (*p<*0.05) (Table 2). Pups in the OZS group had better appetites after 4–5 days’ zinc supplementation. At the end of the experiment, serum zinc concentration and gain of body weight in OZS and CO pups were similar (*p>*0.05). However, pups in the IZS group did not have any improvement in appetites after zinc intravenous injection and still had significantly lower gain of body weight compared with the CO groups (*p<*0.05) although zinc serum concentration of IZS pups was a little higher than that of the ICO group (*p<*0.05) (Table 2).

**Table 2:** The effect of zinc supplementation on serum zinc and gain of body weight in rats

Rats	Group	Serum zinc (µg/l)	Gain of body weight (g)
Maternal	CO (6)	1038.50±68.87	70.50±5.96
	PF (6)	998.67±75.34	61.67±9.09∇
	ZD (6)	657.83±60.83*	12.26±1.45 *
	OZS (6)	661.00±61.88*	10.83±1.94 *
	IZS (6)	684.33±21.12*	12.05±2.44 *
	ICO (6)	659.50±59.94*	11.83±2.34 *
	*F* values	54.80	211.37
	*p* values	<0.001	<0.001
Pups	CO (12)	1427.50±155.38	55.50±9.29
	PF (12)	1376.83±156.67	46.35±8.64∇
	ZD (12)	568.86±57.93*	10.33±2.35*
	OZS (12)	1508.75±157.13	59.42±9.27
	IZS (12)	1843.75±341.33*▼	17.83±6.27*▼
	ICO (12)	559.14±67.71	10.30±1.68
	*F* values	101.96	129.09
	*p* values	<0.001	<0.001

Maternal rats’ serum zinc was measured at postnatal day 21, while pups’ serum zinc was measured at postnatal day 35. Data are expressed as the mean±standard deviation. CO=control; PF=pair fed; ZD=zinc deprived; OZS=oral zinc supplementation; IZS=injection zinc supplementation; ICO=injection control; ()=number of rats.

Comparison between two groups:

* compared with CO and PF, *p*<0.05

∇ compared with CO, *p*<0.05

▼ compared with ICO, *p*<0.05.

### Learning and reference memory test

**Fig. 2:**
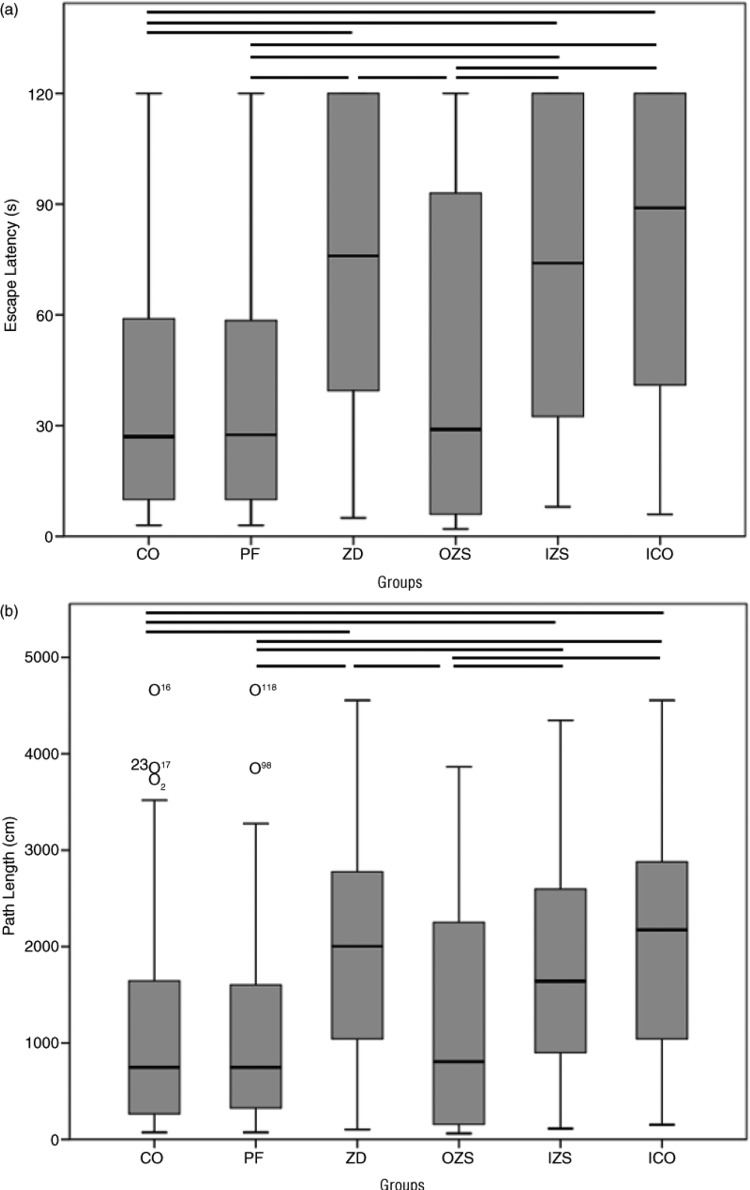
Box plots showing median Escape Latency (a) and Path Length (b) for each group for 4 days in Morris water maze test. Boxes show interquartile ranges, and I bars represent highest and lowest values. The horizontal lines indicate significant comparisons at post-hoc analysis. CO=control; PF=pair fed; ZD=zinc deprived; OZS=oral zinc supplementation; IZS=injection zinc supplementation; ICO=injection control.

**Fig. 3:**
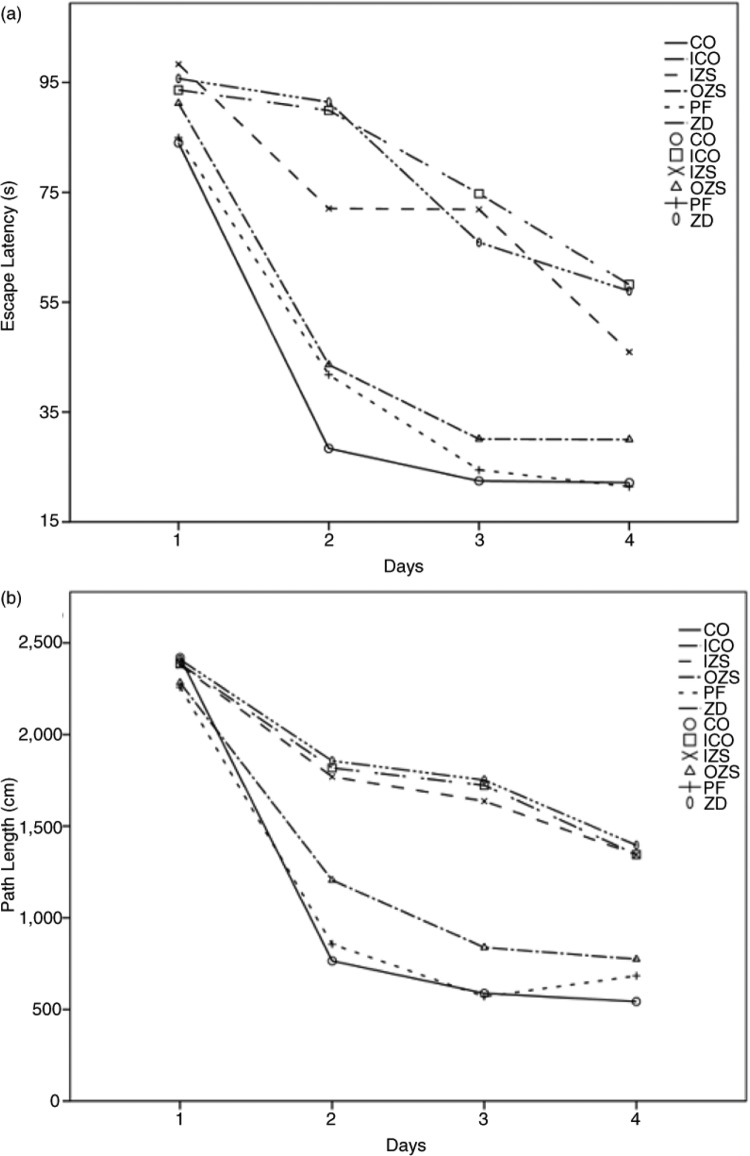
The escape latency (a) and Path length (b) for each animal during each training day. Data are means for 12 pups/group. From the second training day, the changes in the ZD, IZS, and ICO groups were not as obvious as those in the PF, CO, and OZS groups (*p*<0.05). CO=control; PF=pair fed; ZD=zinc deprived; OZS=oral zinc supplementation; IZS=injection zinc supplementation; ICO=injection control.

Compared with the CO and PF groups, the rats in the ZD group were poor in orientation ability, and their swimming traces were distracted, spending more time finding the latent platform (escape latency) and swam a longer path length (*p<*0.05). The indexes in the OZS group were similar to those in the CO group, while the indexes in the IZS group were similar to those in the ZD and ICO groups (*p>*0.05) (Fig. 2). With the increase of training days, both the escape latency and path length were gradually reduced in each group. However, from the third training day, the changes in the ZD, IZS, and ICO groups were not as obvious as those in the PF, CO, and OZS groups (*p*<0.05) (Fig. 3).

## Discussion

In this study, we used a mild dietary zinc deficiency and two different ways of zinc supplementation in rat models to examine changes in learning and memory behavior. However, the effects of zinc deprivation could be ascribed to reduced food intake rather than zinc deficiency. In order to eliminate the above problem, one group of controls (PF group) was fed the same amount of food consumed by the ZD group. The PF rats showed no changes in the parameters assessed, indicating that the effects were specific to zinc deficiency and not the consequence of a general reduction in food intake.

It is well established that mild zinc deprivation during pregnancy and lactation inhibits the growth of pups (23). Consistent results were observed in our study. The growth retardation may be induced by decreased protein synthesis and increased catabolic response to zinc deficiency (24). In our experiments on zinc supplementation, pups of the OZS group were fed an oral zinc–abundant diet, and as a result serum zinc concentration and body weight were significantly improved and caught up with those of the CO group on day 35. The results are consistent with other research outcomes (11, 20), suggesting that adequate OZS after weaning could improve growth of pups. However, few data are available on the effect of injected zinc supplementation after long-term zinc deficiency during pregnancy and lactation on pups’ growth. In the present study, the two different ways of zinc supplementation we selected are consistent with the zinc supplementation ways used clinically (14, 18, 19). The dose of zinc injection was zinc 550 µg/kg (equal to 2.5 mg ZnSO_4_) according to the clinical dose proportion of oral and vein zinc supplementation (approximately 3:1). Though serum zinc concentration was evaluated to normal range (760–1,860 µg/l), the body weight of the pups in the IZS group was not significantly improved and they still presented the symptoms of zinc deficiency. Therefore, the two ways of zinc supplementation for zinc-deficient rats were obviously different in their effects on the growth and zinc-deficient symptoms.

The results from the MWM test revealed that zinc-deficient rats exhibited defects in memory behavior. This is consistent with previous reports showing that dietary zinc deficiency appears to damage learning and memory processes (25–27). Previous studies evaluated the effects of maternal zinc deprivation during critical periods of rapid fetal and infant brain growth, which differed from the present study by the degree of zinc deficiency during different periods. Animal research has shown that severe maternal zinc deficiency (zinc<1 µg/g diet) in early pregnancy, a period of fetal organogenesis, results in fetal brain malformation (25). Pregnant animals given severe zinc-deficient diets also exhibited poor performance of shock-induced learning tasks during the last period of gestation (14–20 days) or during lactation, a period which would not affect organogenesis, but would be critical for brain growth (26, 27). However, maternal rats were given mild zinc-deficient diets through gestation and lactation in this study. Results demonstrated signs of memory impairment in zinc-deficient pups, as indicated by prolonged retrieval latencies and increased escape ratios compared with control pups. All these studies showed consistent results despite different designs. Cognitive impairment may be induced by the decrease in brain protein synthesis and the total amount of brain lipids in the pups (25).

The few studies on the effects of zinc oral supplementation on cognitive recovery in ZD animals have reported conflicting results. Halas et al. (11) showed that zinc nutritional insults during the critical period of cerebral growth were not reversed by subsequent zinc supplementation. However, our study found that OZS rescued the cognitive defects observed in zinc-deficient rats, as reported by Piechal and Tahmasebi (12, 28). The discrepancy of these results may be attributed to age differences in the offspring receiving the cognitive tests. Importantly, OZS in children was also in line with related studies: Zinc supplementation of undernourished children improves their developmental quotients, activity patterns, and neuropsychological functions (29). Our finding that OZS is beneficial in reversing hippocampal dysfunction seems to further substantiate the pivotal role played by synaptic zinc in shaping the physiological neurotransmission of this crucial brain region.

Furthermore, the present study showed that pups with injected zinc supplementation could not improve cognitive ability. This result is consistent with that concerning the body weight of the pups treated with injected zinc supplementation, and it indicates that injected zinc supplementation could not produce a marked physiological function in terms of growth and cognition in zinc-deficient rats. The mechanisms to explain this outcome remains unknown. Although the serum zinc concentration in pups with injected zinc supplementation was elevated to normal range (760–1,860 µg/l), the pups’ cognitive ability could not be reversed. The mechanism of this phenomenon is not fully understood at present. We are well aware that further efforts should be made to explore zinc concentration and zinc homeostasis in the hippocampus of the IZS group.

There are limitations worth noting, such as the zinc levels in hippocampus ought to be measured because the hippocampus is involved in cognition and memory formation. Other studies have reported that dietary zinc deficiency can cause a decrease in zinc concentration in the hippocampus (10). In addition, behavioral studies should be performed by a person who is blind to the different treatment groups, which is of great importance for the behavioral tests.

In conclusion, we observed that mild zinc deficiency during pregnancy and lactation leads to the impairment of spatial learning and memory ability in offspring, and that OZS, instead of intravenous transfusion zinc supplementation, could recover the impairment of spatial learning and memory ability. Based on the overall findings of this study, we suggest that OZS be recommended for treating zinc deficiency. The therapeutic effects should be assessed when zinc supplementation is given by way of long-term intravenous alimentation in clinical practice, and concentration of serum zinc is not to be used as the only index to judge zinc nutritional status for patients treated with long-term intravenous alimentation.

## References

[1] Chiplonkar SA, Kawade R (2012). Effect of zinc- and micronutrient-rich food supplements on zinc and vitamin A status of adolescent girls. Nutrition.

[2] Caulfield LE, Zavaleta N, Shankar AH, Merialdi M (1998). Potential contribution of maternal zinc supplementation during pregnancy to maternal and child survival. Am J Clin Nutr.

[3] Gower-Winter SD, Levenson CW (2012). Zinc in the central nervous system: from molecules to behavior. Biofactors.

[4] Fernandes FS, de Souza AS, do Carmo M, Boaventura GT (2011). Maternal intake of flaxseed-based diet (*Linum usitatissimum*) on hippocampus fatty acid profile: implications for growth, locomotor activity and spatial memory. Nutrition.

[5] Assaf SY, Chung SH (1984). Release of endogenous Zn^2+^ from brain tissue during activity. Nature.

[6] Howell GA, Welch MG, Frederickson CJ (1984). Stimulation-induced uptake and release of zinc in hippocampal slices. Nature.

[7] Hardy AB, Serino AS, Wijesekara N, Chimienti F, Wheeler MB (2011). Regulation of glucagon secretion by zinc: lessons from the beta cell-specific Znt8 knockout mouse model. Diabetes Obes Metab.

[8] Perez-Rosello T, Anderson CT, Schopfer FJ, Zhao Y, Gilad D, Salvatore SR (2013). Synaptic Zn^2+^ inhibits neurotransmitter release by promoting endocannabinoid synthesis. J Neurosci.

[9] Shah D, Sachdev HP (2006). Zinc deficiency in pregnancy and fetal outcome. Nutr Rev.

[10] Gao H-L, Zheng W, Xin N, Chi Z-H, Wang Z-Y, Chen J (2009). Zinc deficiency reduces neurogenesis accompanied by neuronal apoptosis through caspase-dependent and -independent signaling pathways. Neurotox Res.

[11] Halas ES, Hunt CD, Eberhardt MJ (1986). Learning and memory disabilities in young adult rats from mildly zinc deficient dams. Physiol Behav.

[12] Piechal A, Blecharz-Klin K, Pyrzanowska J, Widy-Tyszkiewicz E (2012). Maternal zinc supplementation improves spatial memory in rat pups. Biol Trace Elem Res.

[13] Kumar SP, Anday EK (1984). Edema, hypoproteinemia, and zinc deficiency in low-birth-weight infants. Pediatrics.

[14] Bhutta ZA, Nizami SQ, Isani Z (1999). Zinc supplementation in malnourished children with persistent diarrhea in Pakistan. Pediatrics.

[15] Berger MM, Baines M, Raffoul W, Benathan M, Chiolero RL, Reeves C (2007). Trace element supplementation after major burns modulates antioxidant status and clinical course by way of increased tissue trace element concentrations. Am J Clin Nutr.

[16] Berger MM, Soguel L, Shenkin A, Revelly JP, Pinget C, Baines M (2008). Influence of early antioxidant supplements on clinical evolution and organ function in critically ill cardiac surgery, major trauma, and subarachnoid hemorrhage patients. Crit Care.

[17] Young B, Ott L, Kasarskis E, Rapp R, Moles K, Dempsey RJ (1996). Zinc supplementation is associated with improved neurologic recovery rate and visceral protein levels of patients with severe closed head injury. J Neurotrauma.

[18] Cvijanovich NZ, King JC, Flori HR, Gildengorin G, Vinks AA, Wong HR (2015). A safety and dose escalation study of intravenous zinc supplementation in pediatric critical illness. JPEN J Parenter Enteral Nutr.

[19] Weber TR, Sears N, Davies B, Grosfeld JL (1981). Clinical spectrum of zinc deficiency in pediatric patients receiving total parenteral nutrition (TPN). J Pediatr Surg.

[20] Golub MS, Keen CL, Gershwin ME, Hendrickx AG (1995). Developmental zinc deficiency and behavior. J Nutr.

[21] Bruno RS, Song Y, Leonard SW, Mustacich DJ, Taylor AW, Traber MG (2007). Dietary zinc restriction in rats alters antioxidant status and increases plasma F2 isoprostanes. J Nutr Biochem.

[22] Zhang Y, Li F, Cao X, Jin X, Yan C, Tian Y (2009). Forepaw sensorimotor deprivation in early life leads to the impairments on spatial memory and synaptic plasticity in rats. J Biomed Biotechnol.

[23] Chowanadisai W, Kelleher SL, Lönnerdal B (2005). Maternal zinc deficiency reduces NMDA receptor expression in neonatal rat brain, which persists into early adulthood. J Neurochem.

[24] Giugliano R, Millward DJ (1987). The effects of severe zinc deficiency on protein turnover in muscle and thymus. Br J Nutr.

[25] Jiang YG, Fang HY, Pang W, Liu J, Lu H, Ma Q (2011). Depressed hippocampal MEK/ERK phosphorylation correlates with impaired cognitive and synaptic function in zinc-deficient rats. Nutr Neurosci.

[26] Gao H-L, Xu H, Xin N, Zheng W, Chi Z-H, Wang Z-Y (2011). Disruption of the CaMKII/CREB signaling is associated with zinc deficiency-induced learning and memory impairments. Neurotox Res.

[27] Chu Y, Mouat MF, Harris RB, Coffield JA, Grider A (2003). Water maze performance and changes in serum corticosterone levels in zinc-deprived and pair-fed rats. Physiol Behav.

[28] Tahmasebi Boroujeni S, Naghdi N, Shahbazi M, Farrokhi A, Bagherzadeh F, Kazemnejad A (2009). The effect of severe zinc deficiency and zinc supplement on spatial learning and memory. Biol Trace Elem Res.

[29] Gardner JM, Powell CA, Baker-Henningham H, Walker SP, Cole TJ, Grantham-McGregor SM (2005). Zinc supplementation and psychosocial stimulation: effects on the development of undernourished Jamaican children. Am J Clin Nutr.

